# Sodium aescinate promotes apoptosis of pancreatic stellate cells and alleviates pancreatic fibrosis by inhibiting the PI3K/Akt/FOXO1 signaling pathways

**DOI:** 10.3389/fphar.2025.1554260

**Published:** 2025-04-22

**Authors:** Qing-Yun Wang, Bai-Yan Xu, Yi Wang, Yan-Mei Lin, Lin-Fu Zheng, Gang Liu, Da-Zhou Li, Chuan-Shen Jiang, Wen Wang, Xiang-Peng Zeng

**Affiliations:** ^1^ Department of Digestive Diseases, Dongfang Hospital of Xiamen University, School of Medicine, Xiamen University, Fuzhou, China; ^2^ Department of Digestive Diseases, 900th Hospital of PLA Joint Logistic Support Force, Fuzhou, China; ^3^ Department of Digestive Diseases, Fuzong Clinical Medical College of Fujian Medical University, Fuzhou, China; ^4^ Department of Digestive Diseases, Huian County Hospital, Quanzhou, China; ^5^ Department of Gastroenterology, The Affiliated Hospital of Qingdao University, Qingdao, China

**Keywords:** sodium aescinate (SA), pancreatic fibrosis, pancreatic stellate cells (PSCs), PI3K/AKT/FOXO1 signaling pathway, chronic pancreatitis (CP), apoptosis

## Abstract

Chronic pancreatitis (CP) is an inflammatory disease of progressive pancreatic fibrosis, and pancreatic stellate cells (PSCs) are key cells involved in pancreatic fibrosis. To date, there are no clinical therapies available to reverse inflammatory damage or pancreatic fibrosis associated with CP. Sodium Aescinate (SA) is a natural mixture of triterpene saponins extracted from the dried and ripe fruits of horse chestnut tree. It has been shown to have anti-inflammatory and anti-edematous effects. This study aims to explore the therapeutic potential of SA in CP and the molecular mechanism of its modulation. Through *in vivo* animal models and experiments, we found that SA significantly alleviated pancreatic inflammation and fibrosis in caerulein-induced CP mice model. In addition, SA inhibited the proliferation, migration and activation of PSCs as well as promoted apoptosis of PSCs through a series of experiments on cells *in vitro* including CCK-8 assay, Western blotting, immunofluorescence staining, wound-healing assay, Transwell migration assays, flow cytometric analysis, etc. Further RNA sequencing and *in vitro* validation assays revealed that inhibition of the PI3K/AKT/FOXO1 signaling pathway was involved in the SA mediated promotion of PSCs apoptosis, thus alleviating pancreatic fibrosis. In conclusion, this study revealed that SA may have promising potential as therapeutic agent for the treatment of CP, and the PI3K/AKT/FOXO1 pathway is a potential therapeutic target for pancreatic inflammation and fibrosis.

## 1 Introduction

Chronic pancreatitis (CP) is a progressive, irreversible multifactorial fibroinflammatory syndrome, with the predominant pathological change being recurrent acute inflammation that exacerbates pancreatic fibrosis, resulting in pain, dyspepsia, and diabetes (Beyer et al., 2020; Bai et al., 2024; Witt et al., 2007). At present, the clinical treatment of CP is mainly for the symptomatic treatment of pain, exocrine and endocrine insufficiency, etc., and there is a lack of specific drugs to alleviate pancreatic fibrosis (Singh et al., 2019).

Pancreatic fibrosis is a key pathological feature of CP, with pancreatic stellate cells (PSCs) playing a critical role in its development (Bynigeri et al., 2017). In their quiescent state, PSCs primarily store retinoids, exhibiting limited capacity for proliferation and migration (Ferdek and Jakubowska, 2017). However, when activated by pancreatic inflammation, these cells undergo a dramatic transformation, leading to increased proliferation, migration, and the excessive secretion of extracellular matrix (ECM) components and growth factors (Erkan et al., 2012; Xue et al., 2018). This process promotes the progression of pancreatic fibrosis and exacerbates inflammation (Apte et al., 2011).

**FIGURE 1:**
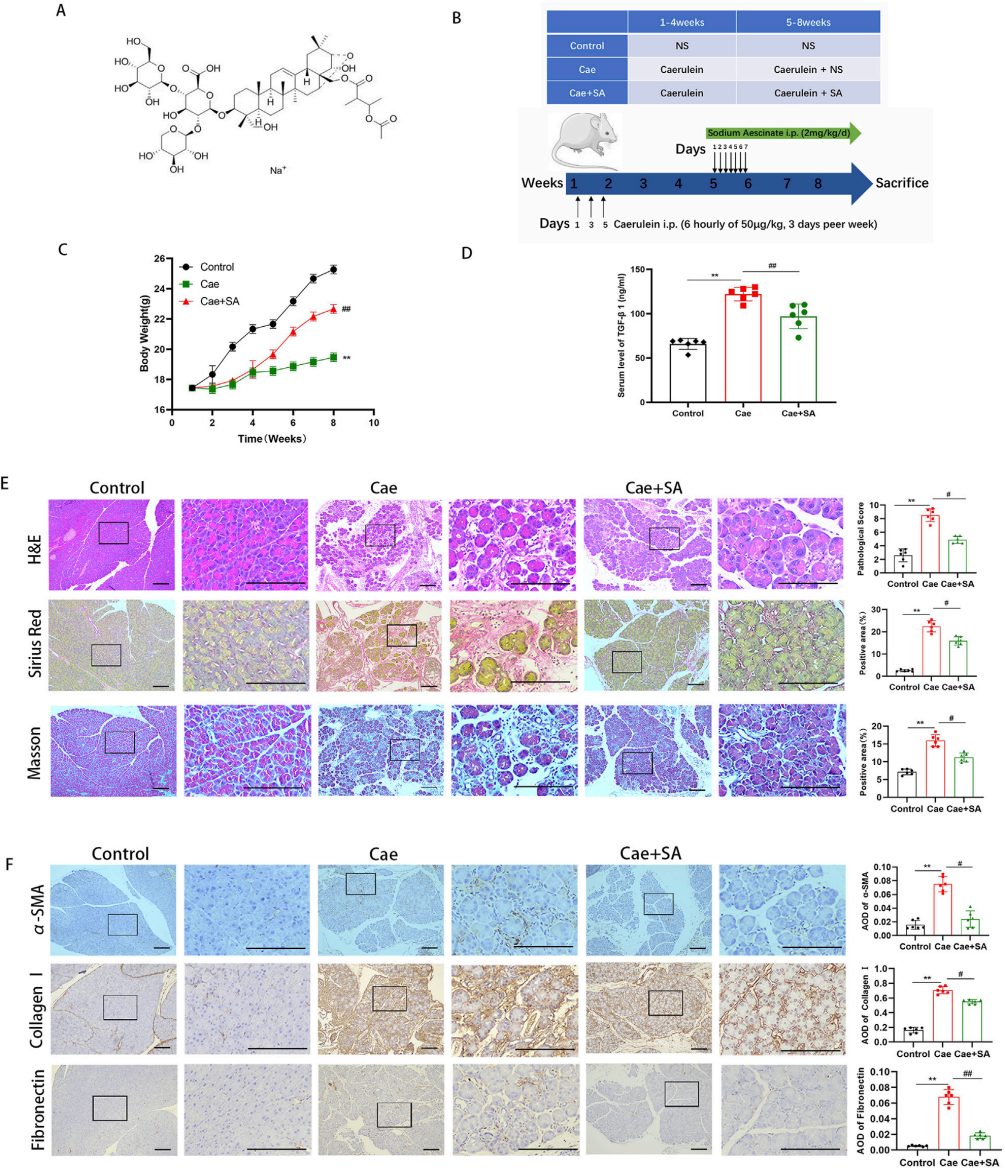
SA *in vivo* alleviated caerulein-induced pancreatic inflammation and fibrosis. **(A)** Schematic diagram of the molecular structure of SA. **(B)** Flowchart of experimental design for cerulein-induced pancreatitis mouse model construction and SA intervention. **(C)** Body weight changes in control group (Control), cerulein-induced model group (Cae), and SA-treated group (Cae + SA) during the experiment (n = 6/group; presented as mean ± SD; ^**^ *P* < 0.01 vs Control group; ^##^ *P* < 0.01 vs Cae group). **(D)** Serum TGF-β1 levels detected by ELISA (n = 6/group, three independent experiments; presented as mean ± SD; ^**^ *P* < 0.01 vs Control group; ^##^ *P* < 0.01 vs Cae group; analyzed by Kruskal–Wallis test and Dunn’s correction). **(E)** Representative images of pancreatic tissue sections stained with H&E, Masson, and Sirius red (scale bar = 100 μm). Right panels show quantitative results of histopathological scores and positive staining area (n = 6; presented as mean ± SD; ^**^ *P* < 0.01 vs Control group; ^#^ *P* < 0.05 vs Cae group; analyzed by Kruskal–Wallis test and Dunn’s correction). **(F)** Immunohistochemical staining of α-smooth muscle actin (α-SMA), fibronectin, and collagen I (scale bar = 100 μm). Bar graphs show mean optical density values (n = 6; presented as mean ± SD; ^**^ *P* < 0.01 vs Control group; ^##^ *P* < 0.01, ^#^ *P* < 0.05 vs Cae group; analyzed by Kruskal–Wallis test and Dunn’s correction).

Sodium Aescinate (SA, molecular structure diagram shown in Figure 1A), the active compound derived from the seeds of the horse chestnut tree, has been recognized for its diverse pharmacological properties (Huang et al., 2022), including anti-inflammatory (Mei et al., 2023), anti-edema (Wang X. et al., 2024), neuroprotective (Chen et al., 2024; Xu et al., 2023; Zhang et al., 2020), antioxidant (Du et al., 2012), and anti-tumor effects (Hou et al., 2019). As a clinical treatment, SA is commonly used to treat cerebral edema, swelling caused by trauma or surgery, and venous return disorders. Xu et al. confirmed that the protection effects of SA on neurons by inhibiting microglia activation through NF-κB pathway (Xu et al., 2023). Furthermore, Li et al. shown that SA can effectively inhibit the proliferation of hepatocellular carcinoma cells by inhibiting the activation of CARMA3/NF-kB signaling in hepatocellular carcinoma (Hou et al., 2019). Although the mechanism of action of SA has been studied more in recent years, studies on its role in ameliorating pancreatic fibrosis in CP are rare.

Cell death occurs through three main programmed pathways: apoptosis, pyroptosis, and necroptosis (Ketelut-Carneiro and Fitzgerald, 2022). Apoptosis, in particular, has long been recognized as the primary regulatory form of cell death and is regulated by a variety of signaling pathways, including the PI3K/AKT pathway (Fresno Vara et al., 2004; Bertheloot et al., 2021). AKT activation promotes cell survival, proliferation, and resistance to apoptosis in various cell types (Brunet et al., 1999). One of the key substrates of AKT is FOXO1, which is a member of the forkhead box O (FOXO) family of transcription factors. Nuclear FOXO1 binds to ligands in the nucleus to inhibit DNA repair and promote apoptosis (Xie et al., 2022). Current studies have shown that activation of the PI3K/AKT pathway leads to an increase in phosphorylated FOXO1, which inhibits the transcriptional function of FOXO1 and contributes to cell survival, growth, and proliferation (Gong et al., 2020). Therefore, dephosphorylated FOXO1 can trigger the expression of target genes that are essential for inducing apoptosis (Li et al., 2023; Luo et al., 2023; Xing et al., 2018). Recent studies have highlighted the association between FOXO1 activity and promotion of apoptosis and attenuation of fibrosis. (Yu et al., 2021; Zhang et al., 2021; Zhang et al., 2024; Qin et al., 2021).

Given these considerations, the aim of this study is to investigate whether SA can attenuate the effects of pancreatic fibrosis and inflammation in mouse models of CP and PSCs. In addition, we aim to elucidate the underlying mechanisms by which SA exerts its role.

## 2 Materials and methods

### 2.1 Animal model setup

Male C57BL/6 mice (6–7 weeks old, 20–22 g body weight) were purchased from Slack Laboratory Animal Co., Ltd. (Shanghai, China) and housed in a specific pathogen-free (SPF) environment. The mice were randomly divided into three groups with 6 mice in each group: Control group, Cae group, and Cae + SA group. Both the Cae group and Cae + SA group were subjected to CP modeling using caerulein. Caerulein was dissolved in saline and administered via intraperitoneal injection on Mondays, Wednesdays, and Fridays each week, with a single injection dose of 50 μg per kilogram of body weight. On each injection day, six consecutive injections were administered at 1-h intervals, and this protocol was maintained for 8 weeks. Control group mice received intraperitoneal injections of an equivalent volume of saline with the same injection volume and frequency as the other two groups. Starting from the 5th week of modeling, the Cae + SA group received intraperitoneal injections of SA for drug intervention. SA was dissolved in saline for administration. Based on references from relevant literature, the single intraperitoneal injection dose of SA in this study was 2 mg per kilogram of body weight, administered once daily at a fixed time (i.e., 2 mg/kg/day). SA was administered during the latter half of the CP modeling period (weeks 5–8) for 4 weeks to observe its effects on pancreatic inflammation and fibrosis. Control group and Cae group mice received intraperitoneal injections of an equivalent volume of saline, with the same administration method as the Cae + SA group. All mice were weighed every Monday before caerulein intraperitoneal injections. On the Wednesday of the 9th week (5 days after the last caerulein intraperitoneal injection for modeling), all mice were uniformly euthanized. This study protocol was approved by the Animal Care Committee of the 900th Hospital of the Joint Logistics Support Force and strictly followed experimental animal ethical standards.

### 2.2 Histology and immunohistochemical staining

Pancreatic tissues from mice were fixed, paraffin-embedded, and sectioned at 4 μm for staining. For H&E staining, sections underwent deparaffinization, rehydration, hematoxylin (3–8 min) and eosin (1–3 min) staining, followed by dehydration and mounting. Sirius Red staining was performed by incubating sections with Sirius Red dye (1 h), followed by washing and dehydration; collagen-positive areas were quantified using ImageJ. Masson’s trichrome staining included sequential staining with Weigert’s iron hematoxylin (nuclei), Ponceau S (cytoplasm), phosphomolybdic acid (differentiation), and aniline blue (collagen), with dehydration and mounting. For IHC, antigen retrieval was performed using citrate buffer under heat. Endogenous peroxidase was blocked with 3% H_2_O_2_. Sections were incubated with primary antibodies (α-SMA 1:500, Collagen I 1:200, Fibronectin 1:400) for 1 h, followed by HRP-conjugated secondary antibodies and DAB chromogen. Nuclei were counterstained with hematoxylin. All sections were dehydrated, mounted, and imaged under a light microscope.

### 2.3 Cell culture and treatment

Human pancreatic stellate cells (PSCs) were provided by Prof. Logsdon (University of Texas MD Anderson Cancer Center). These cells were isolated from pancreatic adenocarcinoma samples using outgrowth techniques and were immortalized. PSCs were cultured in Dulbecco’s Modified Eagle Medium (Hyclone Laboratories, United States), supplemented with 15% fetal bovine serum (Gibco, Thermo Fisher Scientific, United States) and 1% penicillin-streptomycin (Hyclone Laboratories, United States) in a humidified incubator at 37°C with 5% CO_2_. For experiments, when the cells reached 70% confluence, they were treated with varying concentrations of SA (Wuhan ChemFaces Biochemical Co., Ltd.), recombinant human transforming growth factor-β1 (TGF-β1) (MedChemExpress, United States), or the PI3K activator 740 Y-P (MedChemExpress, United States). Western blotting and immunofluorescence analyses were performed on cells during their logarithmic growth phase.

### 2.4 Enzyme-linked immunosorbent assay

Blood samples were collected from the mice, and serum was separated by centrifugation at 3,000 rpm for 15 min and stored at −80°C. Serum levels of TGF-β1 were measured using a commercial mouse TGF-β1 Enzyme-Linked Immunosorbent Assay (ELISA) kit (Boster, Wuhan, China).

### 2.5 Cell viability assay

Cell viability was assessed using the CCK-8 kit. PSCs were seeded in 6-well plates at a density of 1 × 10^6^ cells/well and treated with varying concentrations of SA (0, 20, 40 μM) for 24 or 48 h. After treatment, 10 μL of CCK-8 reagent was added to each well, followed by incubation at 37°C for 2 h. Absorbance at 450 nm was measured using a microplate reader. Results were derived from three independent experiments, each performed in triplicate.

### 2.6 Western blotting analysis

Total protein from PSCs was extracted using RIPA lysis buffer (Beyotime Biotechnology, China), and protein concentration was measured with a BCA assay kit. Equal amounts of protein (20 μg) were separated by SDS-PAGE on 10% gels and transferred to PVDF membranes, which were then blocked with 5% BSA in TBST. Membranes were incubated overnight at 4°C with primary antibodies, followed by HRP-conjugated secondary antibodies for 1.5 h at room temperature. Protein bands were visualized using a chemiluminescent imaging system, with GAPDH serving as a loading control. The primary antibodies used are listed in Table 1.

**TABLE 1:** Primary antibodies used for Western blotting analysis.

Antibody	Host	Type	Company	Dilution
α-SMA	Rb	IgG	Cell Signaling Technology (#19245)	1:1000
Fibronectin	Rb	IgG	Cell Signaling Technology (#26836)	1:1000
Collagen I	Rb	IgG	Cell Signaling Technology (#72026)	1:1000
PI3K	Rb	IgG	Abcam (#ab191606)	1:1000
p-PI3K	Rb	IgG	Affinity (#AF3241)	1:1000
AKT	Rb	IgG	Cell Signaling Technology (#4691)	1:1000
p-AKT	Rb	IgG	Cell Signaling Technology (#4060)	1:1000
FOXO1	Rb	IgG	Cell Signaling Technology (#2880T)	1:1000
p-FOXO1	Rb	IgG	Cell Signaling Technology (#9461T)	1:1000
ERK	Rb	IgG	Cell Signaling Technology (#4695)	1:1000
p-ERK	Rb	IgG	Cell Signaling Technology (#4370)	1:2000
p38MAPK	Rb	IgG	Cell Signaling Technology (#8690)	1:1000
p-p38MAPK	Rb	IgG	Cell Signaling Technology (#4511)	1:1000
Bcl-2	Ms	IgG1	Cell Signaling Technology (#15071)	1:1000
Bax	Rb	IgG	Cell Signaling Technology (#5023)	1:1000
Cleaved Caspase-3	Rb	IgG	Cell Signaling Technology (#9654)	1:1000
Caspase-3	Rb	IgG	Cell Signaling Technology (#9662)	1:1000
GAPDH	Ms	IgG1	Abcam (#ab8245)	1:10000

### 2.7 Immunofluorescence staining

Cells were fixed in 4% paraformaldehyde for 30 min, permeabilized with 0.2% Triton X-100 for 10 min, and blocked with 2% BSA for 1 h. Overnight incubation at 4°C was done with primary antibodies, followed by 1 h at room temperature with Alexa Fluor 488 or Alexa Fluor 594-conjugated secondary antibodies. DAPI was used to stain nuclei, and fluorescent images were captured using an Olympus fluorescence microscope.

### 2.8 Wound-healing assay

PSCs were seeded in 6-well plates (5 × 10^5^ cells/well) and cultured in a 5% CO_2_ incubator at 37°C. Once confluence was reached, a sterile pipette tip created a scratch. After washing with PBS, cells were incubated in serum-free medium with SA (20 or 40 μM) or control medium. The wound area was observed 24 h later using an inverted microscope.

### 2.9 Cell migration assay

Transwell migration assays were performed by seeding PSCs in the upper chamber of the insert (1 × 10^4^ cells/well) in serum-free medium with varying SA concentrations (0, 20, 40 μM). Medium containing 10% FBS was added to the lower chamber. After 24 h at 37°C, non-migrated cells were removed, and the migrated cells were fixed in paraformaldehyde, stained with crystal violet, and counted under a light microscope.

### 2.10 Flow cytometric analysis

PSCs were seeded in 6-well plates (1 × 10^6^ cells/well) and treated with SA (0, 20, 40 μM) for 48 h. Following trypsinization and centrifugation, cells were resuspended in binding buffer containing propidium iodide (PI) and annexin V-FITC for analysis via flow cytometry.

### 2.11 TUNEL assay

PSCs treated with SA, with or without the PI3K activator 740 Y-P, were fixed, permeabilized, and subjected to TUNEL staining to evaluate apoptosis. Cells were incubated with TUNEL reaction mixture at 37°C for 1 h, then visualized under a fluorescence microscope.

## 3 Statistical analysis

In this study, GraphPad Prism 9.0.0 software was used for statistical analysis. All 3 independent experiments were completed for all content, and all data were presented as mean ± standard deviations. The Shapiro-Wilk test for normality was used for normality and the Brown-Forsythe test for homogeneity of variance. The unpaired t-test was used to evaluate the significance between the two groups. The paired t-test is used to compare the differences between paired samples. For comparisons of more than three groups, analysis of variance was used. Use nonparametric statistical tests for non-normal data or small sample sizes.Statistical significance was defined as *p* < 0.05 and the chart was presented as a bar chart marked with significance (^*^ *P* < 0.05, ^**^ *P* < 0.01, ^#^ *P* < 0.01, ^##^ *P* < 0.01).

## 4 Results

### 4.1 SA *in vivo* alleviated caerulein-induced pancreatic inflammation and fibrosis

To establish an experimental chronic pancreatitis (CP) model, male C57BL/6 mice were administered caerulein (Cae, 50 μg/kg) via intraperitoneal injection every 6 h, 3 days per week, for a total duration of 8 weeks. From the 5th week to the end of the 8th week (28 days in total), mice received daily injections of SA (2 mg/kg), as shown in Figure 1B. Compared to the control group, mice in the Cae group exhibited a significant reduction in body weight during the experiment (*P* < 0.01, Figure 1C). However, compared to the Cae group, body weight in the Cae + SA group partially recovered (*P* < 0.01), suggesting that repeated caerulein injections significantly reduced body weight, an effect that was alleviated by SA treatment. Meanwhile, serum TGF-β1 levels, a key marker of fibrosis, were significantly elevated in the Cae group compared to the control group but were markedly reduced in the Cae + SA group compared to the Cae group (Figure 1D). To further confirm the therapeutic effects of SA, histopathological analyses of pancreatic tissues were conducted using H&E staining, Masson’s trichrome staining, and Sirius red staining. The results demonstrated that key pathological features of CP, including acinar cell atrophy, extracellular matrix (ECM) protein deposition, ductal dilation, and immune cell infiltration, were significantly alleviated in the Cae + SA group compared to the Cae group (Figure 1E). Additionally, immunohistochemical analysis revealed that, compared to the control group, the expression levels of fibrosis markers α-SMA and ECM proteins (Fibronectin, Collagen I) were significantly elevated in pancreatic tissues of the Cae group. However, following SA treatment, these fibrosis markers were significantly reduced compared to the Cae group (Cae + SA group vs Cae group, *P* < 0.05, Figure 1F).

### 4.2 SA inhibited the proliferation, migration and activation of PSCs

**FIGURE 2:**
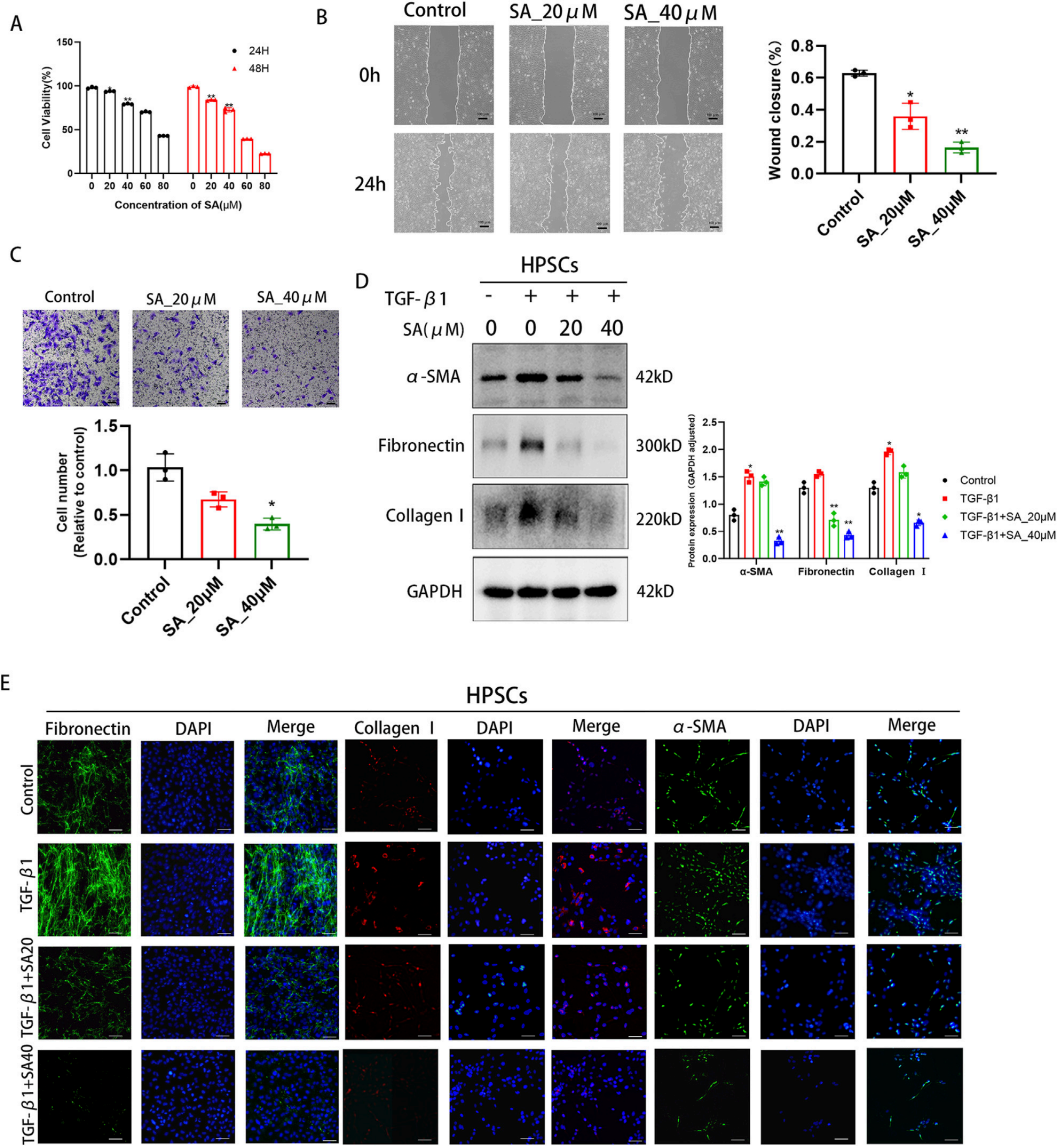
SA inhibited the proliferation, migration and activation of PSCs. **(A)** The effects of different concentrations of SA (0, 20, 40, 60, 80 μM) on PSC viability after 24/48 h treatment were detected by CCK-8 assay (^*^ *P* < 0.05, ^**^ *P* < 0.01 compared to 0 μM group, presented as mean ± SD, n = 3/group, three independent experiments). **(B)** Scratch assay evaluated PSC migration ability after 24 h SA treatment (0, 20, 40 μM). Scratch areas were quantified using ImageJ (n = 3 fields/group, three independent experiments, presented as mean ± SD; ^*^ *P* < 0.05, ^**^ *P* < 0.01 compared to control group). **(C)** Transwell assay detected the number of migrated PSCs after 24 h SA treatment (0, 20, 40 μM) (^*^ *P* < 0.05 compared to control group, three independent experiments, presented as mean ± SD). **(D)** Western blot analysis of α-SMA, Collagen I, and Fibronectin expression in PSCs treated with different SA concentrations for 24 h before and after TGF-β1 activation (GAPDH as loading control, n = 3, ^*^ *P* < 0.05, ^**^ *P* < 0.01 compared to control group by Paired t-test, presented as mean ± SD). **(E)** Immunofluorescence staining showing the effects of SA intervention on Fibronectin (green), Collagen I (red), and α-SMA (green) expression in TGF-β1-activated PSCs. Nuclei were stained with DAPI (blue, scale bar = 100 μm).

As illustrated in Figure 2A, the viability of PSCs progressively declined with rising doses of SA over time. To minimize potential cytotoxic effects, a dose of 40 μM (yielding approximately 80% cell viability) was selected as the maximum administered concentration for subsequent experiments. PSCs activation is also characterized by migratory behavior. By using SA in the scratch wound healing assay, we found that the migration area of PSCs into the wound area was significantly reduced with increasing SA concentration in a dose-dependent manner (Figure 2B). Similarly, in the transwell migration assay, SA greatly reduced the amount of PSCs crossing the PET membrane (Figure 2C). These findings confirmed the significant inhibitory effect of SA on the migration of PSCs. TGF-β1 is the most potent fibrogenic cytokine known to date. TGF-β1 significantly stimulates the activation and proliferation of PSCs. We conducted Western blotting analyses on PSCs treated with SA, both in the presence and absence of TGF-β1. The protein levels of fibrosis markers like Collagen I, Fibronectin and the cellular activation marker, α-SMA, decreased in a dose-dependent manner as SA concentrations increased (Figure 2D). To further validate the inhibitory impact of SA on PSCs activation, we performed immunofluorescence staining for each of the markers: Collagen I, Fibronectin and α-SMA. As shown in Figure 2E, protein expression levels significantly declined with increasing doses of SA combined with TGF-β1. These data indicate that the SA effectively suppressed PSCs activation and the production of ECM proteins.

### 4.3 RNA sequencing analysis of PSCs treated with SA

**FIGURE 3:**
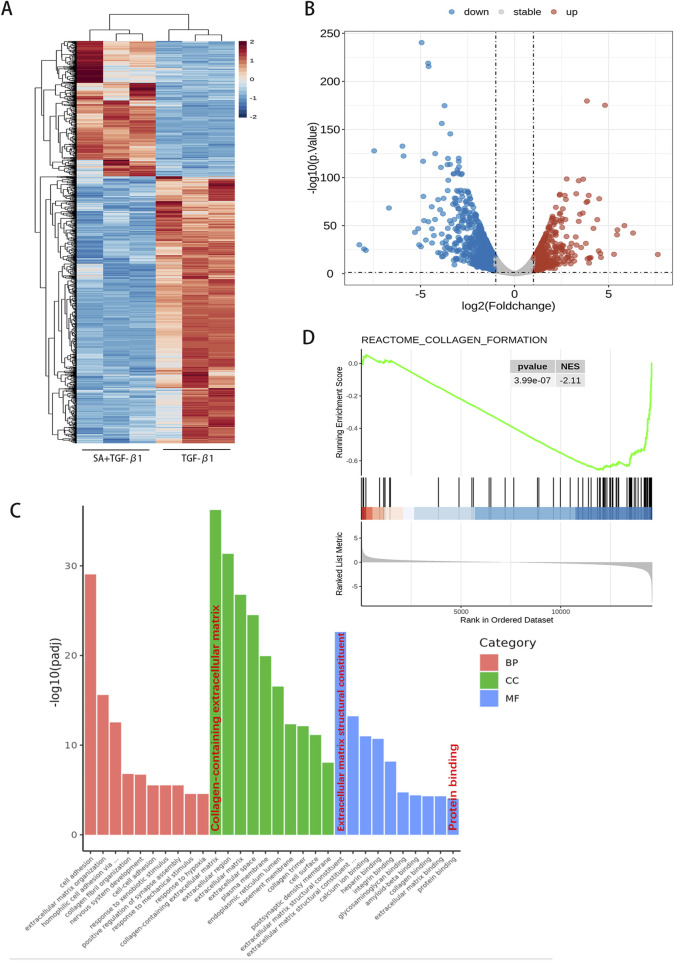
RNA sequencing analysis of PSCs treated with SA. Using the screening criteria (*P* < 0.05 and fold change >2 or <0.5), a total of 1,610 differentially expressed genes (DEGs) were identified. **(A)** A hierarchical clustering heatmap and **(B)** volcano plot illustrate the gene expression profiles. **(C)** Gene Ontology (GO) functional enrichment analysis based on hypergeometric distribution and **(D)** Gene Set Enrichment Analysis (GSEA, analyzed using R software, n = 3).

**FIGURE 4:**
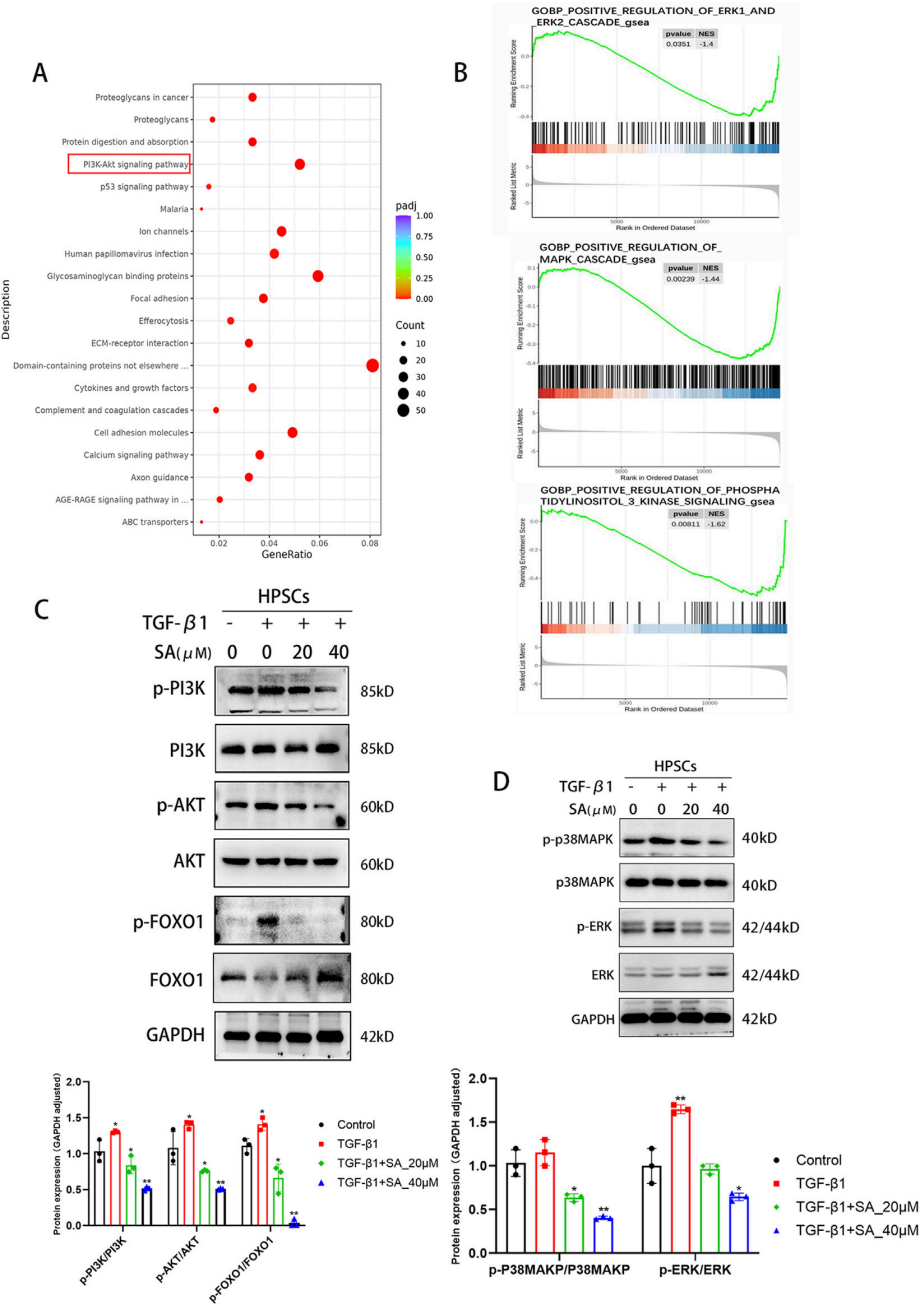
SA inhibited the activation and proliferation of PSCs through the PI3K/AKT Signaling Pathway and MAPK Signaling Pathway. **(A)** KEGG pathway enrichment and **(B)** GO functional enrichment analyses revealed key signaling pathways associated with DEGs. **(C)** Western blot analysis of total and phosphorylated protein levels of PI3K, AKT, and FOXO1 in PSCs treated with SA before and after TGF-β1 activation (n = 3, ^*^ *P* < 0.05, ^**^ *P* < 0.01 compared to control group by Paired t-test, presented as mean ± SD). **(D)** Changes in MAPK pathway protein expression (ERK1/2, p38-MAPK) following SA intervention, quantified by bar graphs (n = 3, presented as mean ± SD; ^*^ *P* < 0.05, ^**^ *P* < 0.01 compared to control group by Paired t-test).

To further explore the molecular mechanisms by which SA affects PSCs, we performed RNA sequencing analysis on PSCs exposed to SA (40 μM, 24 h) + TGF-β1 and PSCs activated by TGF-β1 alone (Figure 3). Using screening criteria of P < 0.05 and a fold change greater than 2, we identified 1,610 differentially expressed genes (DEGs), consisting of 541 upregulated and 1,069 downregulated genes, between the SA + TGF-β1 and TGF-β1 groups. Figures 3A, B display a hierarchical clustering heatmap and a volcano plot of the DEGs, respectively. Gene ontology (GO) enrichment analysis (Figure 3C) indicated that these DEGs were mainly associated with processes related to extracellular matrix (ECM) regulation, cell adhesion, and other significant pathways. Furthermore, Gene Set Enrichment Analysis (GSEA) showed that collagen formation-related genes, including collagen I, were significantly downregulated in the SA + TGF-β1 group, which corroborates the results from the Western blotting analysis (Figure 3D). KEGG pathway enrichment analysis of the DEGs (Figure 4A) highlighted several critical pathways, including the PI3K-AKT and p53 signaling pathways. Further gene ontology enrichment analysis (Figure 4B) indicated enrichment in the PI3K signaling, MAPK cascades, and ERK1/2 pathways. The transcription factor FOXO1, which regulates cell growth, metabolism, and apoptosis, is influenced by the upstream PI3K/AKT pathway. Western blotting analysis revealed that with higher doses of SA in the presence of TGF-β1, the phosphorylated levels of PI3K, AKT, FOXO1, ERK1/2, and p38 MAPK significantly decreased, while the total protein levels of FOXO1 decreased and the total protein levels of PI3K, AKT, ERK1/2, and p38 MAPK remained unchanged (Figures 4C, D). These findings suggest that the inhibitory effects of SA on the activation and proliferation of PSCs are mediated through the suppression of the PI3K/AKT and MAPK signaling pathways.

### 4.4 SA promoted the apoptosis of PSCs via inhibiting PI3K/AKT/FOXO1 signaling pathway

**FIGURE 5:**
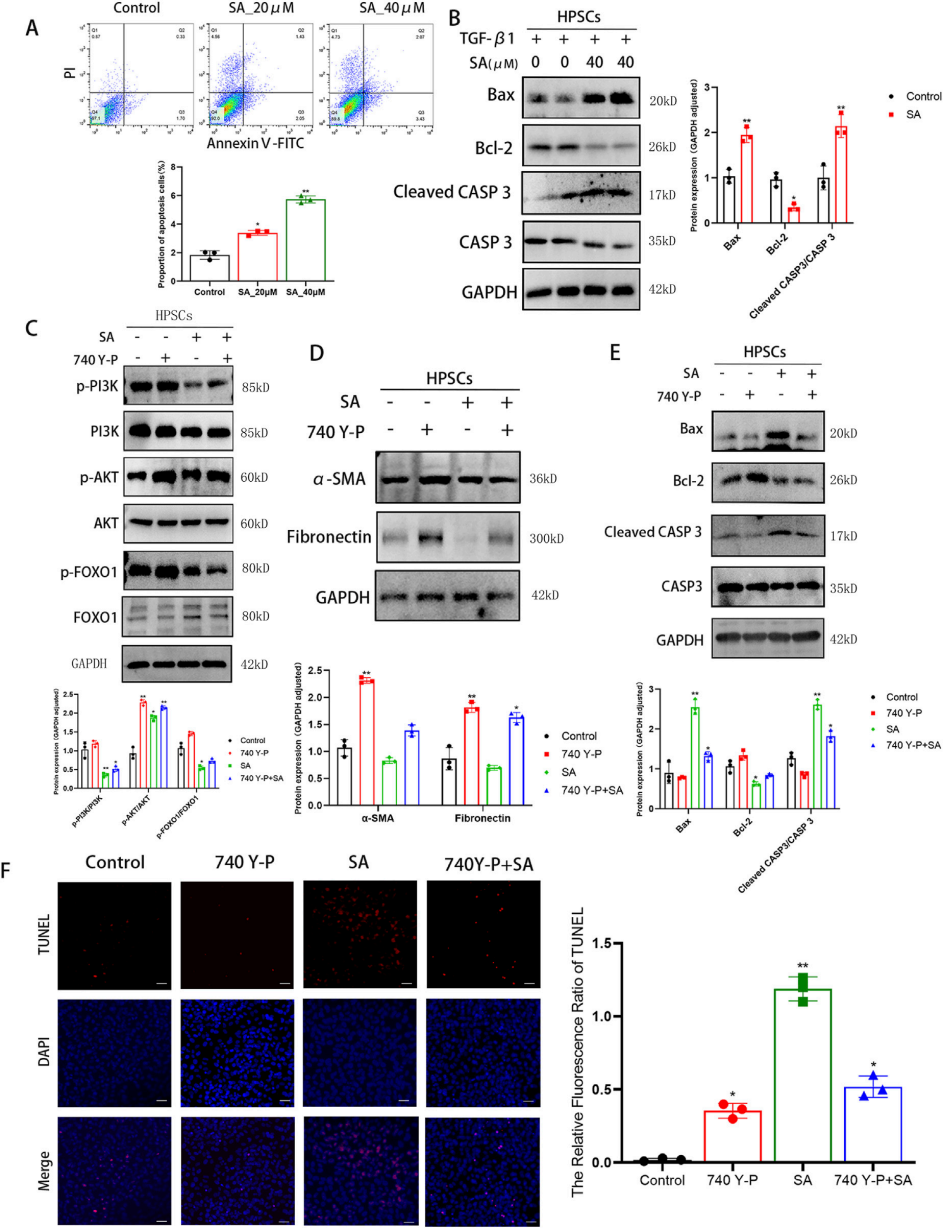
SA promoted the apoptosis of PSCs via inhibiting PI3K/AKT/FOXO1 signaling pathway. **(A)** Apoptosis rates (early + late apoptosis) of PSCs treated with SA for 48 h were measured by Annexin V/PI double staining flow cytometry (n = 3, ^*^ *P* < 0.05, ^**^ *P* < 0.01 vs control group. presented as mean ± SD). **(B)** Western blot analysis of apoptosis-related protein expression in PSCs treated with TGF-β1 combined with SA for 24 h (GAPDH as loading control, n = 3, ^*^ *P* < 0.05,^**^ *P* < 0.01 compared to control group by Paired t-test, presented as mean ± SD). **(C)** Validation of total and phosphorylated protein levels of PI3K, AKT, and FOXO1 under different treatment conditions (n = 3, ^*^ *P* < 0.05, ^**^ *P* < 0.01 compared to control group by Paired t-test, presented as mean ± SD). **(D, E)** Expression levels of α-SMA, Fibronectin **(D)** and apoptosis-related proteins **(E)** across experimental groups (n = 3, ^*^ *P* < 0.05, ^**^ *P* < 0.01 compared to control group by Paired t-test, presented as mean ± SD). **(F)** TUNEL fluorescence staining showing cellular apoptosis (scale bar = 100 μm, n = 3, ^*^ *P* < 0.05, ^**^ *P* < 0.01 vs. control; presented as mean ± SD).

To examine the pro-apoptotic effects of SA on PSCs, flow cytometry and Western blotting analyses were performed. As shown in Figure 5A, SA significantly increased apoptosis in a dose-dependent manner. Additionally, SA treatment upregulated the levels of pro-apoptotic proteins Bax and cleaved Caspase 3 (CASP3) without affecting total CASP3 levels, while the anti-apoptotic protein Bcl-2 was downregulated (Figure 5B). To further investigate the molecular mechanism behind SA-induced apoptosis and its anti-fibrotic effects, we used the PI3K activator 740 Y-P. Western blotting results demonstrated that 740 Y-P restored the phosphorylation of PI3K, AKT, and FOXO1 in SA-treated cells, while the total protein level of FOXO1 decreased, but PI3K and AKT total protein levels remained unaffected (Figure 5C). Additionally, 740 Y-P increased the expression of α-SMA and Fibronectin (Figure 5D). Furthermore, 740 Y-P reversed the upregulation of Bax and cleaved CASP3 induced by SA, while restoring Bcl-2 levels (Figure 5E). TUNEL assays also confirmed that 740 Y-P alleviated SA-induced apoptosis (Figure 5F). In conclusion, SA promotes apoptosis and reduces fibrosis in PSCs by modulating the PI3K/AKT/FOXO1 signaling pathway.

**FIGURE 6:**
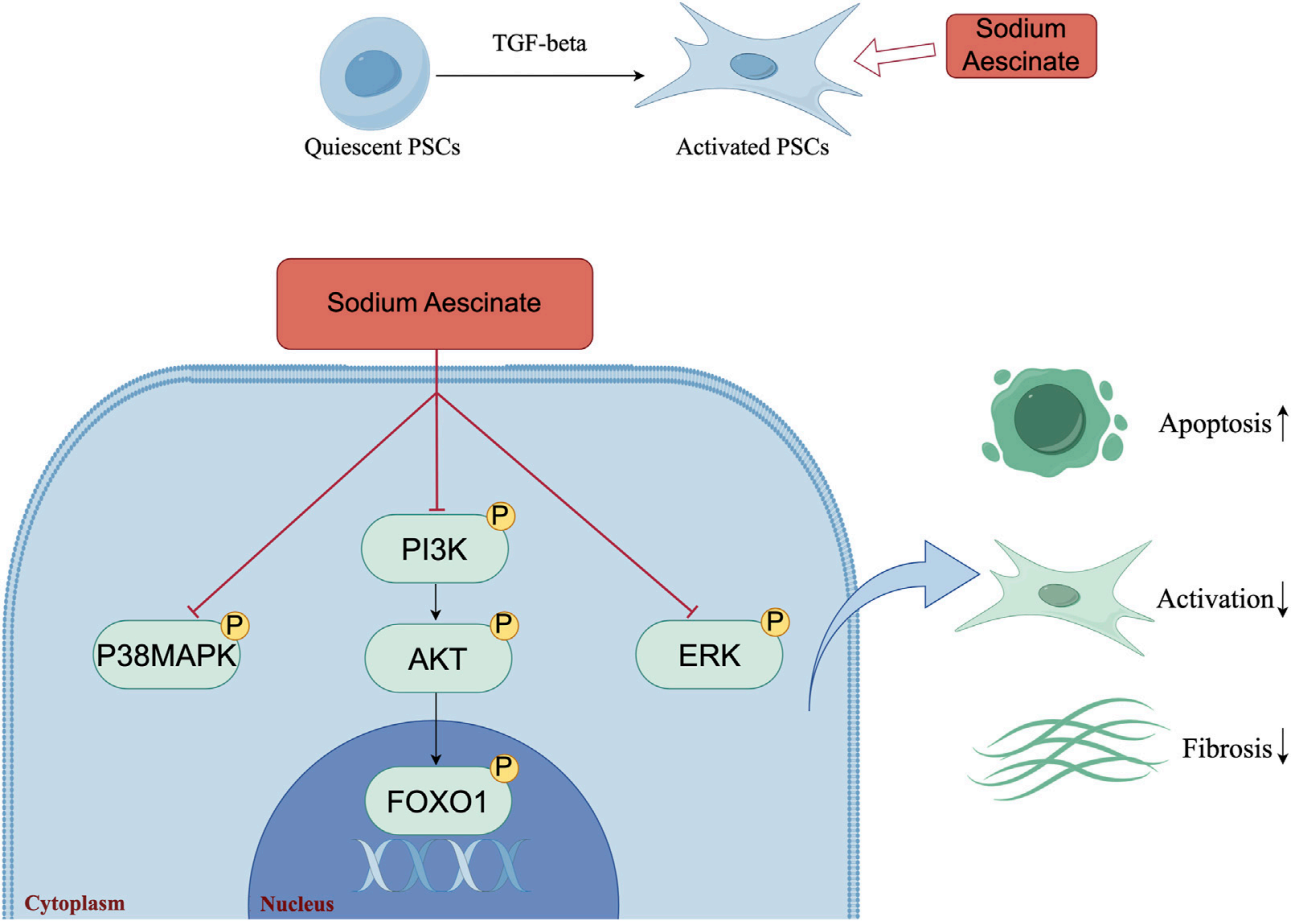
The Mechanism Diagram of the Article (by Figdraw). The schematic model illustrated the underlying mechanism by which SA induced the apoptosis of PSCs, inhibited the activation of PSCs and panreatic fibrosis. The figure visually showed the key molecular events and pathways involved in SA-induced apoptosis.

## 5 Discussion

This study is the first to explore the effects of SA on the inhibition of the fibrotic phenotype in PSCs and its underlying mechanisms. Previous studies have shown that SA can inhibit microglia activation and neuroinflammation through selective inhibition of the JNK/p38 signaling pathway and binding to NF-kappaB signaling pathway proteins, thereby exerting neuroprotective effects. Besides, SA can reduce the development of fibrosis by regulating the expression of M1-type macrophage cytokines, showing great potential in the treatment of lymphedema (Wang X. et al., 2024; Xu et al., 2023; Xie et al., 2024). SA is usually widely used in clinical practice (Hokkoku et al., 2024; Xia et al., 2020; Zeng et al., 2021; Tao et al., 2020). However, its role and mechanism to attenuate pancreatic fibrosis as well as inflammation in CP have not been reported so far. In our research, we found that SA *in vivo* alleviated caerulein-induced pancreatic inflammation and fibrosis and *in vitro* inhibited the proliferation, migration and activation of PSCs. Furthermore, SA induced apoptosis and reduced ECM production in PSCs via the PI3K/AKT/FOXO1 signaling pathway. From the above results, we concluded that SA promoted the apoptosis of PSCs through regulating PI3K/AKT/FOXO1 pathway, then inhibiting the activation of PSCs and pancreatic inflammation and fibrosis in progression of CP.

We first assessed the severity of chronic pancreatitis induced by repeated caerulein injections, a widely utilized experimental model for CP (Sun et al., 2017; Pan LL. et al., 2023; Zhang et al., 2017; Choi et al., 2020). Compared to the Control group, mice in the Cae group exhibited significant weight loss, elevated serum TGF-β levels, and histopathological features (e.g., acinar atrophy and inflammatory cell infiltration) in H&E staining, confirming successful model establishment. In contrast, the Cae + SA group showed markedly reduced fibrosis compared to the Cae group, as evidenced by Sirius Red staining, Masson’s trichrome staining, and immunohistochemical analysis, collectively supporting the therapeutic efficacy of SA.

We then investigated the effect of SA on PSCs (the primary effector cells responsible for driving pancreatic fibrosis). Following pancreatic injury and inflammation, PSCs transition from a quiescent to an activated state, where they secrete large quantities of ECM components, such as collagen, connexins, various cytokines and chemokines (Auwercx et al., 2025; Yang et al., 2016). This process contributes to the development of pancreatic fibrosis, severely impairing the normal function of the pancreas (Li et al., 2018). Numerous studies have focused on mitigating pancreatic fibrosis by inhibiting the overactivation of PSCs (Tamura et al., 2021; Xue et al., 2023; Szabó et al., 2024). In our study, scratch and migration assays demonstrated that SA effectively inhibited both the proliferation and migration of PSCs in a dose-dependent manner. TGF-β1 is a potent activator of PSCs, and recent studies have optimized stimulation concentrations to induce PSCs activation (Wang Y. et al., 2024). When treated with SA after activation, Western blotting analysis revealed a dose-dependent reduction in the activation of PSCs. These results suggested that SA could significantly alleviate PSCs activation, offering potential therapeutic benefits in pancreatic fibrosis.

Next, we further investigated the mechanism of anti-activation effect of SA on PSCs by RNA sequencing. The MAPK cascade is a central signaling pathway that regulates a variety of stimulated cellular processes, including proliferation, differentiation, apoptosis, and stress responses (Plotnikov et al., 2011). Previous studies have found that MAPK signaling is involved in pancreatic fibrosis by regulating PSCs functions, such as PSCs proliferation, migration, and apoptosis (Jin et al., 2020). In activated PSCs, phosphorylation of ERK and p38 MAPK was overexpressed (Xu et al., 2018; Sun et al., 2024). In our study, the results of Western blotting showed that SA exerted significant inhibitory effects on both p-ERK and p-p38 MAPK of activated PSCs. These results supported the inhibition of SA on the activation of PSCs. Previous researchers have found SA inhibits the proliferation of Y79 cells by arresting the cell cycle at the G2/M phase, and induces apoptosis via the caspase-related apoptosis pathway (Li L. et al., 2020). SA inhibited the proliferation and induce apoptosis in the gastric cancer BGC-823 cells and AGS cells, and this process can be implemented by inhibiting the JAK-1/STAT-1 signaling pathway (Zhong, 2016). SA triggers hepatocyte ferroptosis by inhibiting the activity of ATF4, resulting in an oxidative imbalance (Xi et al., 2025). And SA could block signals transiting to downstream molecules AKT, ERK, inhibit the proliferation of breast cancer cell MCF-7 cell apoptosis and induced cell apoptosis by suppressing the activation of SRC (Qi et al., 2015). However, whether SA promotes the apoptosis of PSCs and thus alleviates pancreatic fibrosis has not been reported. In the present study, we found that SA promoted apoptosis of PSCs via experimental techniques including the flow cytometry and Western blotting. Previous studies have found that PI3K/AKT/FOXO1 is involved in the regulation of apoptosis (Pan Z. et al., 2023; Li K. et al., 2020). Liang et al. found that MiR-224-5p overexpression upregulates the expression of the antioxidant gene SOD2 through the PI3K/Akt/FOXO1 axis to relieve H/R-induced oxidative stress and reduce apoptosis of H9c2 cells (Liang et al., 2024). In our study, to assessed whether the PI3K/AKT/FOXO1 pathway influences SA-induced apoptosis in PSCs, we employed the classical PI3K activator, 740Y-P (Wang S. et al., 2024; Mo et al., 2024; Zou et al., 2024). Western blotting analysis revealed that activation of the PI3K/AKT/FOXO1 pathway by 740Y-P reversed the inhibitory effects of SA. Previous researches have established that the TUNEL assay detects DNA fragmentation by labeling the free 3ʹ-hydroxyl ends, a process that occurs during both early and late stages of apoptosis. As such, TUNEL staining remains a widely used indicator for detecting apoptotic cell death (Mirzayans and Murray, 2020; Loo, 2011). In comparison to the group treated solely with SA, the 740Y-P + SA group exhibited a significant reduction in TUNEL-positive cells. Simultaneously, levels of the pro-apoptotic proteins Bax and Cleaved CASP3 decreased, while the anti-apoptotic protein Bcl-2 increased. These findings demonstrate that activation of the PI3K/AKT/FOXO1 pathway by 740Y-P can effectively counteract SA-induced apoptosis. Moreover, further analysis showed that markers of fibrosis, such as α-SMA and ECM protein fibronectin, were elevated in the 740Y-P + SA group compared to the SA group, indicating an enhanced activation of PSCs. These results suggested a link between PSCs activation and apoptosis regulation via the PI3K/AKT/FOXO1 pathway.

Although this study revealed the ameliorative effects of SA on chronic pancreatitis-associated fibrosis, several limitations remain. First, while pancreatic stellate cell culture systems are widely used as *in vitro* models for screening antifibrotic drugs, the simplified monoculture environment fails to recapitulate the complex cellular interactions and dynamic drug metabolism *in vivo*. For instance, although the experimental SA dose (2 mg/kg) showed efficacy in the mouse model, it differs from the clinical dosage of sodium aescinate (5–20 mg/day in humans). The clinical dosage is primarily used to alleviate edema and venous insufficiency; however, its role in pancreatic fibrosis requires further validation. Future studies should integrate organoid co-culture systems or *in situ* models (e.g., precision-cut pancreatic slices) to better mimic the human drug response microenvironment. Second, the effects of SA on other CP-associated cell types (e.g., pancreatic acinar cells, immune cells) remain unexplored, limiting our understanding of its global therapeutic mechanisms. Finally, the translational gap between animal models and clinical application needs to be bridged. For example, the clinical safety, long-term efficacy, and dose optimization of SA require validation through multicenter clinical trials to advance its translation from preclinical research to therapeutic practice.

In conclusion, this study comprehensively investigated that SA could attenuate pancreatic fibrosis through *in vivo* and *in vitro* experiments. Mechanistically, SA could promote apoptosis of PSCs through inhibiting PI3K/AKT/FOXO1 signaling pathway, while significantly inhibiting the proliferation and activation of PSCs (Figure 6). These results indicated that SA may have promising potential as therapeutic agent for the treatment of CP, and the PI3K/AKT/FOXO1 pathway is a potential therapeutic target for pancreatic inflammation and fibrosis.

## Data Availability

The raw data supporting the conclusions of this article will be made available by the authors, without undue reservation.
